# The Impact of Past Trauma on Psychological Distress: The Roles of Defense Mechanisms and Alexithymia

**DOI:** 10.3389/fpsyg.2020.00992

**Published:** 2020-05-21

**Authors:** Siqi Fang, Man Cheung Chung, Yabing Wang

**Affiliations:** ^1^Department of Social and Behavioural Sciences, City University of Hong Kong, Hong Kong, China; ^2^Department of Educational Psychology, Faculty of Education, The Chinese University of Hong Kong, Shatin, Hong Kong

**Keywords:** past trauma, posttraumatic stress disorder, alexithymia, defense mechanisms, psychological distress

## Abstract

**Objectives:**

Posttraumatic stress disorder (PTSD) symptoms following past trauma could lead to psychological distress. Little is known, however, about the roles of defense mechanisms and alexithymia may play in the process. The current study aimed to examine the potential impact of alexithymia and defense mechanisms on the relationship between past trauma and distress among Chinese university students.

**Method:**

455 university students completed a set of questionnaires: PTSD Checklists for DSM-5, Toronto Alexithymia Scale (TAS-20), Defense Style Questionnaire, and General Health Questionnaire-28.

**Results:**

PTSD following past trauma was associated with increased psychological distress. Alexithymia and defenses (especially immature defense) mediated the path between PTSD and psychological co-morbidities.

**Conclusion:**

Following past trauma, people developed PTSD and other psychological symptoms. The severity of these distress symptoms was influenced by the way they defended themselves psychologically, and their ability to identify, express, and process distressing emotions.

## Introduction

Exposure to a traumatic event is a statistically normative experience in the general population, as exemplified by large scale epidemiology studies (Kilpatrick et al., 2013; Benjet et al., 2016), many of which (approximately 56–74%) have reported exposure to at least one potentially traumatic event (e.g., assault or accident) during their lives (Norris, 1992; Kessler et al., 1995; Breslau, 2002; Atwoli et al., 2015). Increasing evidence from research and clinical practice shows that a history of trauma is a potent risk factor for psychological disorders (Davidson et al., 1991; Halligan and Yehuda, 2000; Keane et al., 2006) of which posttraumatic stress disorder (PTSD) is the most common (APA, 2013).

Notwithstanding this, following a trauma, not all victims will go on to develop PTSD; some may experience disruptions in functioning but gradually return to normal, others maintain clinically significant psychological co-morbidities (e.g., Smyth et al., 2008) such as depression (Hill, 2003; Briere, 2004) and anxiety (Heim and Nemeroff, 2001) which require prolonged intervention (Kessler et al., 2005; Espié et al., 2009; Bonanno and Mancini, 2012). Individual differences suggest that the impact of traumatic life events on distress outcomes is not a direct one (Verhaeghe and Vanheule, 2005) but through a variety of factors. The current research endeavored to understand the mediating mechanisms that link PTSD from past trauma with psychological distress by focusing on two potential psychodynamic constructs: ego defense mechanisms and alexithymia.

Psychological defenses following traumas aim to hide or alleviate conflicts or stressors that give rise to anxiety (Beresford, 2012). It is an automatic and unconscious psychological process that aims to protect the individual against psychological distress and prevent awareness of internal or external danger and stress (Vaillant, 1971, 1992). Although it has been removed from the Diagnostic and Statistical Manual of Mental Disorders (5th ed; APA, 2013), its clinical utility and predictive value for positive adaptation is profound (Vaillant, 2011). Defenses may be ordered hierarchically and divided into four categories according to their psychosocial maturity and level of adaptiveness (Vaillant, 1992): (1) psychotic defenses (e.g., psychotic denial and distortion) which aim to avoid or circumscribe conflicts encountered in the relationship with the external world; (2) immature defenses (e.g., acting out, passive aggression, and projection) which aim to lessen distress and anxiety caused by threatening individuals or uncomfortable reality; (3) neurotic defenses (e.g., displacement, isolation, and repression) which aim to keep potentially threatening feelings, ideas, memories, wishes, or fears out of awareness (Bond, 1986); and (4) mature defenses (e.g., sublimation, humor, and altruism) which may maximize gratification and allow more conscious awareness of feelings, ideas, and their consequences (Vaillant, 2011).

Defense style may be an important factor in explaining the aforementioned individual differences in trauma reactions (Jun et al., 2015). Some defenses are more associated with psychiatric symptoms than others (Jun et al., 2015). For example, psychotic and immature defenses have been found in PTSD victims of different types including combat veterans (Silverstein, 1996; Vaillant, 2011). Abundant studies have also reported that immature defense styles were associated with distress and psychological symptoms (Nickel and Egle, 2006; Diehl et al., 2014; Martino et al., 2020) such as anxiety and depressive disorders (Bond, 2004) among people with a history of childhood trauma and major illness. Other findings also demonstrated that immature defense mediated the relationship between childhood trauma and psychopathology in adulthood (Finzi-Dottan and Karu, 2006; Nickel and Egle, 2006). As such, it is reasonable to speculate that the development of psychological problems after trauma may be mediated by a specific type of defense mechanism. However, to the best of our knowledge, no study has investigated whether defenses might mediate the impact of PTSD from past trauma onto psychological distress among non-clinical samples (Jun et al., 2015).

The stress response syndrome (Horowitz, 1976) emphasizes the need for trauma victims to make the internal model of psychic trauma coherent with existing mental schemas (the completion tendency). However, the trauma-related information may be so incompatible with the existing schema of the world and themselves that it creates overwhelming emotional distress. To prevent emotional exhaustion, an inhibitory mechanism may be activated to inhibit intensive and negative emotions (Helmes et al., 2008; Meganck et al., 2013) and avoid frightening or intolerable feelings (Busch, 2014). This response aims to keep not only distressing emotions at bay but also the individual from integrating the traumatic experience with long-term meaning representations (Horowitz, 1986).

The emergence of alexithymia can be seen as one of these unconscious inhibitory responses which is in itself a defense mechanism (Kooiman et al., 1998). Coined by Nemiah and Sifneos (1970), “*alexithymia*” is defined as having difficulty identifying and describing feelings, relying instead on external oriented thinking (EOT; Taylor et al., 1997). It has been postulated that victims subconsciously adopt alexithymia to cut off overwhelming emotional distress by preventing themselves from accessing internal feelings (Ahrens and Deffner, 1986; Helmes et al., 2008; Meganck et al., 2013; Chung et al., 2016) but focusing on external facts (e.g., Gross, 1998; Kupchik et al., 2007; Declercq et al., 2010). However, unresolved and unprocessed feelings accumulated in the body can cause later disturbance in the physiological and neurological system, giving rise to health difficulties and a range of psychological distress symptoms (e.g., Pennebaker and Beall, 1986; Karukivi et al., 2010; Marchini et al., 2018; Martino et al., 2019a). It is therefore unsurprising that alexithymia often negatively correlates with physical and psychological well-being, but positively associates with physical illness, somatization (Taylor et al., 1991; Sayar et al., 2003), anxiety (Karukivi et al., 2010), and depression (Honkalampi et al., 2000). As such, alexithymia may act as another mediator in the relationship between PTSD from past trauma and psychological distress. This has received support from several studies (Helmes et al., 2008; Chung et al., 2013; Chung and Hunt, 2014; Chen and Chung, 2016).

As was mentioned, since alexithymia can function as a defense, it is not surprising that it has been correlated with increased use of defenses in several studies (e.g., Besharat and Shahidi, 2011; Evren et al., 2012). Specifically, some studies found that alexithymia was positively associated with immature defenses among samples from different countries (Wise et al., 1991; Parker et al., 1998). Similarly, alexithymia showed moderate positive correlations with neurotic defense among both clinical and non-clinical samples (e.g., Parker et al., 1998); this result is not always consistent however (e.g., Bogutyn et al., 1999). Although research has demonstrated that alexithymia was negatively correlated with mature defenses among psychiatric outpatients (Kooiman et al., 1998), a recent study showed that alexithymia and emotional suppression (as a mature defense) “independently” mediated the path between PTSD from past trauma to distress outcomes (Chung and Hunt, 2014). On the contrary, another finding showed that suppression as a defense did not mediate outcomes in conjunction with alexithymia among asthma sufferers (Chung et al., 2012). In short, evidence on the interlocking relationship between alexithymia and defenses is inconsistent.

**FIGURE 1:**
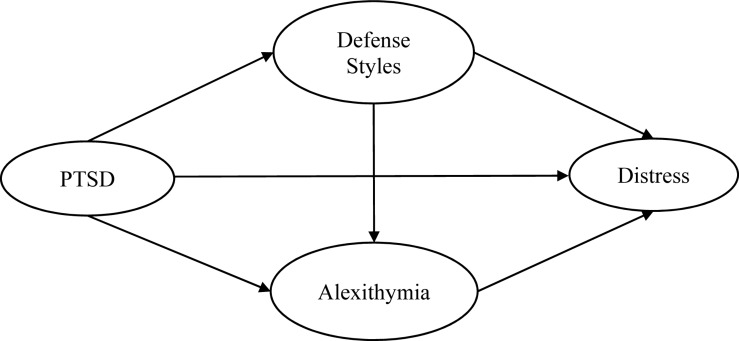
Theoretical model. PTSD = posttraumatic stress disorder symptoms, Distress = psychological distress.

The current study aimed to examine a theoretical framework demonstrating the potential mechanisms of defense styles and alexithymia mediating the impact of PTSD from past trauma onto psychological distress. This framework is shown in Figure 1. Our hypotheses were as follows: (1) PTSD following past trauma would be positively associated with psychological distress; (2) alexithymia and defense style would mediate the links between PTSD and distress outcomes respectively; and (3) alexithymia would be significantly positively associated with defense style.

## Materials and Methods

### Participants and Procedure

Approval was obtained from the ethics committee at the affiliated university (approval number: EDU2016-0218). Four hundred and fifty-five university students from Mainland China and Hong Kong participated in this study. Participants were recruited via advertisements posted in student resident halls and email via the university mass mailing system. The inclusion criteria were: (1) university students with an active student registration status, (2) over 18, (3) no history of major physical illness, and (4) no psychiatric history. A briefing page stated that the study aimed to examine the association among traumatic life events, emotion, psychological defense, and mental health. Students were informed that the study was entirely voluntary and anonymous. Data would be kept confidential. Participants were not financially compensated and entitled to exit from the study at any point.

### Measures

#### Demographic Information

Participants provided information on age, gender, marital status, study mode, and ethnicity.

#### Alexithymia

Toronto Alexithymia Scales (TAS-20, Bagby et al., 1994) is a 20-item self-report questionnaire on a five-point Likert scale from “1 = Never” to “5 = Very often.” It measures the degree of alexithymia with three dimensions: (1) difficulties in identifying feelings (DIF); (2) difficulties in describing feelings (DDF); and (3) externally oriented thinking (EOT). The reliability of subscales was found to be satisfactory (DIF: 0.86; DDF: 0.71; and EOT: 0.61) and a 3-week test–retest reliability was 0.77 in previous research (Chung and Wall, 2013). Cronbach’s alphas of current study for DIF, DDF, and EOT were 0.84, 0.70, and 0.60.

#### Defense Styles

Defense Style Questionnaire-40 (DSQ-40, Andrews et al., 1993) aims to measure the hierarchy of defense mechanisms indicating the individual’s typical mode of dealing with conflict (Vaillant, 1977). It is a 40-item questionnaire with a nine-point Likert scale (1 = strongly disagree to 9 = strongly agree) measuring three types of defense styles (immature, neurotic, and mature). Sample items are “I often act impulsively when something is bothering me” (immature style), “If someone mugged me and stole my money, I’d rather he be helped than punished” (neurotic style), and “I work out my anxiety through doing something constructive and creative like painting or wood-work” (mature style). The construct validity has been demonstrated among different clinical populations and test–retest correlations were also reported with an average *r* of 0.66 using a 4-week time period (Andrews et al., 1993). Cronbach’s alpha ranged from 0.64 to 0.83 (Sinha and Watson, 1999) and were reported as 0.73, 0.70, and 0.88, respectively, for immature, neurotic, and mature defenses, respectively, in this study.

#### PTSD

Posttraumatic stress disorder Checklist for DSM-5 (PCL-5, Weathers et al., 2013) aims to measure posttraumatic stress reactions following a traumatic event. Part I assesses the trauma exposure in the past and lists 17 categories of traumatic event, such as natural disaster, motor vehicle accident, assault, and sudden death of a loved one. Participants indicated whether they had experienced, witnessed, or never been exposed to them. If they experienced more than one trauma, they proceeded to indicate which trauma was the most traumatic. Part II is a 20-item self-report measure designed to assess the PTSD symptoms (intrusion, avoidance, negative mood and cognition, and hyperarousal) using the rating scale 0 = not at all to 4 = extremely. Cronbach’s alphas were 0.93, 0.90, 0.93, and 0.93, respectively, for the current study.

#### Psychiatric Co-Morbidity

General Health Questionnaire-28 (Goldberg and Hillier, 1979) is a 28-item scale on a four-point Likert scale (0 = better than usual to 3 = worse than usual) measuring four types of health conditions (somatic symptoms, anxiety and insomnia, psychosocial dysfunction, and severe depression). Numerous studies have shown that the reliability was excellent as α ranging from 0.90 to 0.95 (Sterling, 2011). Cronbach’s alphas were reported as 0.82, 0.90, 0.81, and 0.93 for each dimension for the current study.

### Data Analysis

Descriptive statistics were used to describe demographic information and the prevalence rate of PTSD. Correlation coefficients were used to establish whether demographic variables were related to distress outcomes. Multivariate normality was checked and no variables were needed for transformation. No outliers were detected during the exploration of diagnostics (Mahalanobis distance = 3 SD). Assumptions relating to linearity, and homoscedasticity were met. Regression imputation was used to address missing data. With less than 2% of responses missing, as in this case, there is little distortion (Schafer and Graham, 2002).

To test our theoretical model (see Figure 1), the following steps were taken. First, we conducted a confirmatory factor analysis to assess the measurement model (MM). Second, the model was tested with structural equation modeling (SEM) (Arbuckle, 2012) to examine whether alexithymia and each defense style would mediate the relationships between PTSD from past trauma and distress outcomes. Maximum likelihood (ML) estimation was adopted. The evaluation of the overall model fit was based on these indices: chi-square (χ^2^) statistics, comparative fit index (CFI), Tucker–Lewis index (TLI), and root-mean-square error of approximation (RMSEA), and 90% confidence intervals. For CFI and TLI, values greater than.90 indicate acceptable model fit. For RMSEA, a value less than 0.08 reflects reasonable model fit (Hu and Bentler, 1998). Data were analyzed using SPSS 25.0 version and AMOS 24.0.

Furthermore, we used PROCESS (Hayes, 2016) to verify the mediational effects of the hypothesized model. Bias-corrected bootstrapping was adopted to generate confidence intervals which addressed the problem of power resulting from the asymmetric and non-normal sampling distributions of the indirect effect (MacKinnon et al., 2004). Point estimates and 95% confidence intervals were estimated for the indirect effects. When zero was not contained in the confidence interval, point estimates of indirect effects were then considered to be significant.

## Results

### Descriptive and Correlation Results

All 455 participants (M = 170, 37%, F = 285, 63%) were full time students, not married, and ranged from 18 to 25 years old (M = 19.75, SD = 1.67). Among them, 65% (*n* = 295) reported having experienced at least one traumatic life event, of whom 11% experienced only one, 18% two, 10% three, and 26% four or more (LEC total, M = 4.52, SD = 6.12). Using the DSM-5 diagnostic criteria, we found that 23% (*n* = 105) and 36% (*n* = 165) met the criteria for full and partial PTSD, respectively. The rest had no PTSD (41%, *n* = 185). No PTSD referred to participants who did not meet any of the diagnostic criteria for the four clusters of PTSD symptoms. Partial-PTSD referred to those who had met the diagnostic criteria for one or two of the symptom clusters (Chung and Wall, 2013). There were no significant correlations between demographic variables and distress outcomes (gender, *r* = −0.002, *ns*; age, *r* = 0.028, *ns*). Table 1 shows the results of means, standard deviations (SDs), and Pearson correlations for all observed indicators.

**TABLE 1:** Means, standard deviations, and bivariate correlations of indicators.

**Indicator**	**1**	**2**	**3**	**4**	**5**	**6**
1. PTSD	—					
2. Alexithymia	0.31**	—				
3. Immature	0.31**	0.34**	—			
4. Neurotic	0.09	0.02	0.64**	—		
5. Mature	0.05	−0.04	−0.61**	0.69**	—	
6. Distress	0.49**	0.35**	0.35**	0.07	0.03	—
Mean	12.08	52.07	114.60	40.44	43.07	52.42
Standard deviation	15.55	10.34	24.03	9.31	8.80	13.02
Range	0–60	20–80	24–216	8–72	8–72	28–112

*p < 0.05, **p < 0.01.

### Measurement Model (MM)

All the indicators loaded significantly on their factors, indicating that the variables had a good validity, except EOT. As noted in Section “Measures,” this subscale demonstrated low internal consistency. Similar results were also reported in previous studies (Mazzeo and Espelage, 2002; Mazzeo et al., 2008; Fang and Chung, 2019). Consequently, we dropped the indicator of EOT from the MM. Table 2 shows information on the Goodness of fit of the MM indicating acceptable fit of the data.

**TABLE 2:** Fit indices for measurement model and structural models.

**Models**	**χ^2^**	**df**	**CMIN/df**	**CFI**	**TLI**	**NFI**	**RMSEA**	**90% CI for RMSEA**
Measurement model	234.79	83	2.83	0.96	0.95	0.94	0.06	0.05, 0.07
Structural model (SM1, immature)	178.38	70	2.59	0.97	0.96	0.95	0.06	0.05, 0.07
Structural model (SM2, neurotic)	204.74	70	2.93	0.96	0.95	0.94	0.07	0.06, 0.08
Structural model (SM3, mature)	227.39	70	3.25	0.95	0.94	0.93	0.07	0.06, 0.08

### Structural Models

**FIGURE 2:**
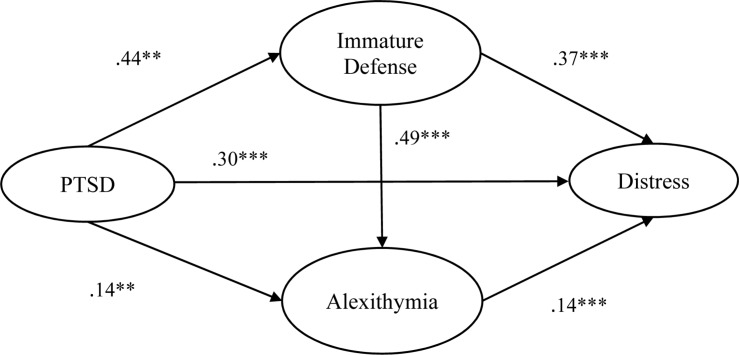
Structural model and SEM coefficients. **p < 0.01, ***p < 0.001. PTSD = posttraumatic stress disorder symptoms, Distress = psychological distress.

For Structural Model 1 (SM1) with immature defense as the index defense, all the coefficients of latent variables reached significance (*p* < 0.05) ranging from 0.14 to 0.49 (see Figure 2). For Structural Model 2 (SM2) and Structural Model 3 (SM3) with neurotic and mature defenses as index defenses, respectively, alexithymia was significantly correlated with PTSD and distress (see Tables 3, 4). Neurotic defense was also significantly correlated with PTSD. Otherwise, it was not associated with distress or alexithymia. Mature defense was not correlated with any of the other latent variables. Results on model fit indicated acceptable fit (see Table 2).

**TABLE 3:** Unstandardized and standardized factor loadings of the hypothesized measurement model.

**Parameter estimate**	**Unstandardized**	**Standardized**
**Measurement model**		
PTSD → Hyperarousal	1.00	0.88***
PTSD → Intrusion	0.92 (0.034)	0.88***
PTSD → Avoidance	0.62 (0.027)	0.80***
PTSD → Negative mood and cognition	1.19 (0.037)	0.96***
Alexithymia → DIF	1.00	0.98***
Alexithymia → DDF	0.50 (0.045)	0.70***
Immature defense → Parcel 1	1.00	0.70***
Immature defense → Parcel 2	0.86 (0.073)	0.65***
Immature defense → Parcel 3	1.03 (0.084)	0.68***
Immature defense → Parcel 4	0.93 (0.071)	0.73***
Mature defense → Sublimation	1.00	0.61***
Mature defense → Humor	1.10 (0.114)	0.66***
Mature defense → Anticipation	1.00 (0.108)	0.62***
Mature defense → Suppression	1.11 (0.113)	0.69***
Neurotic defense → Undoing	1.00	0.63***
Neurotic defense → Pseudo	1.06 (0.117)	0.73***
Neurotic defense → Idealization	0.99 (0.119)	0.54***
Neurotic defense → Reaction formation	0.74 (0.095)	0.48***
Distress → Somatic	1.00	0.76***
Distress → Anxiety	1.43 (0.082)	0.87***
Distress → Social dysfunction	0.55 (0.060)	0.45***
Distress → Depression	1.25 (0.078)	0.73***

***p < 0.001. PTSD = posttraumatic stress disorder symptoms, Neg Mo&Co = negative mood and cognition, Danger = preoccupied with danger, DIF = difficulty in identifying feelings, DDF = difficulty in describing feelings, Passive Agres = passive aggressive, Somatic = somatic symptoms, Anxiety = anxiety and insomnia, Social Dys = psychosocial dysfunction.

**TABLE 4:** Unstandardized and standardized coefficients of the hypothesized structural model.

**Parameter estimate**	**Unstandardized**	**Standardized**
**Structural model**		
PTSD → Immature defense	0.87*** (0.109)	0.44***
PTSD → Mature defense	0.14 (0.083)	0.09
PTSD → Neurotic defense	0.20* (0.086)	0.13*
PTSD → Alexithymia (immature)	0.61** (0.222)	0.14**
PTSD → Alexithymia (mature)	1.56*** (0.212)	0.35***
PTSD → Alexithymia (neurotic)	1.50*** (0.212)	0.33***
PTSD → Distress (immature)	0.02*** (0.004)	0.30***
PTSD → Distress (mature)	0.03*** (0.004)	0.41***
PTSD → Distress (neurotic)	0.03*** (0.004)	0.42***
Alexithymia → Distress (immature)	0.01** (0.001)	0.14**
Alexithymia → Distress (mature)	0.01*** (0.001)	0.32***
Alexithymia → Distress (neurotic)	0.01*** (0.001)	0.31***
Immature defense → Alexithymia	1.10*** (0.135)	0.49***
Mature defense → Alexithymia	0.06 (0.159)	0.02
Neurotic defense → Alexithymia	0.38* (0.16)	0.13*
Immature defense → Distress	0.01*** (0.003)	0.37***
Mature defense → Distress	-0.00 (0.003)	-0.03
Neurotic defense → Distress	0.00 (0.003)	0.01

*p < 0.05, **p < 0.01, ***p < 0.001. PTSD = posttraumatic stress disorder symptoms, Neg Mo&Co = negative mood and cognition, Danger = preoccupied with danger, DIF = difficulty in identifying feelings, DDF = difficulty in describing feelings, Passive Agres = passive aggressive, Somatic = somatic symptoms, Anxiety = anxiety and insomnia, Social Dys = psychosocial dysfunction.

### Direct and Indirect Effects of Alexithymia and Defense Mechanism

Based on the SEM results, we further examined the direct and indirect effects of different defense mechanisms and alexithymia on distress outcomes by computing the multiple mediation bootstrap analysis using PROCESS. The indirect effects of PTSD from past trauma on distress outcomes through immature defenses and alexithymia were significant with confidence intervals being statistically different from zero based on a 95% bootstrap. Also, the direct effect of PTSD on distress remained significant (β = 0.29, *SE* = 0.04, *t* = 8.07, *p* < 0.001). In other words, alexithymia and immature defenses partially mediated the association between PTSD from past trauma and distress (see Table 5).

**TABLE 5:** Total, direct, and indirect effects of PTSD tot (X) on distress (Y) via mediators.

***R* = 0.57, *R*^2^ = 0.33, *F* = 72.96, *p* < 0.001**				
**Total effect of X on Y**				
**Effect**	**SE**	***t***	**LLCI**	**ULCI**
0.41	0.03	11.87	0.34	0.48
**Direct effect of X on Y**				
**Effect**	**SE**	***t***	**LLCI**	**ULCI**
0.29	0.04	8.07	0.22	0.36
**Indirect effect(s) of X on Y via mediators (total score)**				
**Variable**	**Effect**	**SE**	**Lower**	**Upper**

Total	0.12	0.02	0.09	0.16
Immature defenses	0.08	0.02	0.05	0.12
Immature defenses—Alexithymia	0.02	0.01	0.01	0.03
Alexithymia	0.02	0.01	0.01	0.04

*PTSD = posttraumatic stress disorder symptoms, Distress = psychological distress.*

## Discussion

This study explored the interrelationships between PTSD from past trauma, alexithymia, defense mechanisms, and psychological distress. Hypotheses were partially supported. First, PTSD from past trauma was significantly associated with psychological distress. Second, alexithymia and immature defense partially mediated the path between PTSD and distress. Third, alexithymia was positively correlated with immature defense. However, no significant correlation was found between alexithymia, mature, and neurotic defenses.

Consistent with literature (e.g., Vrana and Lauterbach, 1994; Bernat et al., 1998), students developed PTSD after traumatic life events. Although past trauma happened one year ago, on average, approximately 23 and 36% of the sample still experienced full and partial PTSD symptoms, respectively. Further analysis revealed that all PTSD subscales were positively correlated with all psychological co-morbid symptoms ranging from 0.22 to 0.50 in *r* values. This is not surprising given that PTSD is often expressed through other psychological symptoms (Miller et al., 2004).

The prevalence rate for full PTSD was higher than the rates reported in literature ranging from 6 to 17% (e.g., Smyth et al., 2008). This might be due to the different tools used to assess PTSD, as the Impact of Event Scales (IES, Horowitz et al., 1979) were used in Smyth’s study. Furthermore, while intrusive symptoms may be universal, avoidance has been shown to be culturally specific (Marsella et al., 1996; Norris et al., 2001). People with Eastern cultural beliefs may express a higher degree of avoidance and numbness leading to higher PTSD prevalence rates than those with Western cultural characteristics (Foa et al., 1989; Macdonald et al., 2013). However, this interpretation needs to be verified using cross-cultural comparative studies.

The current findings provided further evidence to the relationship between PTSD from past trauma and alexithymia (Bagby and Taylor, 1997; Frewen et al., 2008), between alexithymia and distress (Lumley et al., 2007), and the mediational effect of alexithymia (Helmes et al., 2008; Chung and Hunt, 2014; Chen and Chung, 2016). Students with a high level of trauma severity tended to display a high level of alexithymia which was associated with elevated psychological distress (Zhu et al., 2006). These results have contradicted the claim that alexithymia is an unconscious defense mechanism which acts as a protective strategy by repressing particular stimulations (e.g., Wise et al., 1990; Kooiman et al., 1998). Instead, such inhibitory strategy might be maladaptive in nature and associated with extreme affective impoverishment (Van der Kolk and Ducey, 1989), impacting psychological distress.

In psychoanalytic literature, the inability to put experiences into words and active motivation to forget unacceptable memories leads to repression. Alexithymia may be the presentation of such unconscious repression and acting out (Van der Kolk and Ducey, 1989). As a result, however, traumatized students may continue to experience the effects of the unintegrated experiences and unresolved distressing emotions, until they learn to put into words both the associated facts and the feelings. Accumulated unprocessed distressing feelings are related to disturbances in both physical and psychological health (e.g., Amstadter and Vernon, 2008).

Similarly, traumatized students who used immature defenses tended to report elevated psychological distress. This result echoed previous findings on immature defense mediating the impact of, for example, childhood trauma on psychopathology in adulthood (Finzi-Dottan and Karu, 2006; Nickel and Egle, 2006). It needs to be interpreted with caution, however, because evidence exists to show that immature defenses can be adaptive in certain contexts (Walburg and Chiaramello, 2015). For instance, the adaptive role of denial has been found among individuals with trauma (e.g., Langher et al., 2019) and bereavement experiences especially at the very beginning of their grieving process (Chabrol and Callahan, 2018). Denial can act as a defense against the painful effects of a devastating illness which resembles the process of somatic depersonalization whereby patients detach bodily experiences from the self (Caputo, 2019b). Manic reparation is also thought to be adaptive in facing depressive feelings. The present traumatized students might have relied on it to restore a damaged self by alleviating guilt feelings, enhancing self-efficacy, and preserving hope after trauma (e.g., Caputo et al., 2019). Thus, depending on different contexts in which trauma occurred, students might have benefited from endorsing different immature defenses. But this speculation remains to be investigated.

Immature defense was positively correlated with alexithymia which supported some findings in literature (Wise et al., 1990; Kooiman et al., 1998). Further regression analysis revealed that projection (β = 0.15) and acting out (β = 0.15) were among the strongest predictors to alexithymia. The former is an unconscious desire to displace unwanted or unacceptable feelings onto others while the latter is an attempt to engage in action so that internal feelings are not reflected upon. It is an unconscious expression of an impulse to avoid the awareness of certain affects. In other words, both defenses aim not to confront internal emotions but to deny them. The more students engaged in these specific immature defenses, the more they found difficulty in accessing, articulating and expressing their internal feelings (i.e., alexithymia). This interlocking psychological reaction seemed to have related to the increase of psychological distress for students who had experienced trauma.

This interlocking psychological reaction did not emerge, however, for those who primarily endorsed neurotic and mature defenses. The psychological process whereby alexithymia mediated the impact of past trauma onto distress seems to have occurred independently of the levels of neurotic and mature defenses used. In fact, inconsistent with literature (e.g., Kwon and Lemon, 2000; Kennedy et al., 2001; Watson, 2002; Corruble et al., 2004; Eglinton and Chung, 2011), neurotic and mature defenses did not seem to relate to almost any of the other psychological constructs. In other words, while, according to literature (Walburg and Chiaramello, 2015), it might be beneficial or adaptive to develop greater psychological flexibility of the defense system (i.e., as opposed to relying simply on one defense) in responding to various situations, with defense style correlated with specific psychological distress symptoms or disorder (Akkerman et al., 1992; Bond, 2004; Flett et al., 2005), the present results suggest that some of these defenses might in fact have little impact on distress outcomes. As a result, the notion that all defense styles would influence distress outcomes needs to be revisited.

Nevertheless, these results were mainly based on the relationship between defense mechanisms and distress outcomes. The interactionists (Endler, 1983) would argue that it is an oversight not taking account of environmental or situational factors, personality traits, and cognitive processes (Endler, 1983) which could have produced different results of defense as a coping process for distress. After all, the university students in our samples were mostly “emerging adults,” who, according to Erikson (1979) developmental stages, would have been in a transition stage whereby they have entered into an intellectually stimulating environment at a time when issues pertaining to identity formation are being confronted which would have implications for personality and cognitive developments. These environmental, personality, and cognitive developments could have played a role in influencing the impact of defense on distress outcomes.

Several limitations should be noted before drawing conclusions. First, the use of self-reported measures might have enhanced social desirability and thereby affected the reporting of trauma and well-being measures (e.g., Caputo, 2017). Second, although a mediational relationship was examined in this cross-sectional study, causality between the psychological constructs should be interpreted with caution. We have softened the language by focusing on direct and indirect effects (i.e., structural relationships) rather than causality inference. Nevertheless, our results have set a solid foundation for a future longitudinal study. Third, this study was based on retrospective data, the passage of time and distance from past trauma exposure may have blurred the reporting of PTSD. Fourth, our participants were composed of university students recruited using a convenience sampling method. As a result, this has cast doubt on the generalizability of findings. Other populations will need to be included in future studies. Last but not least, other unobserved variables would have been involved in explaining the mediating effect of current findings. For example, given the low desirability of presenting themselves as traumatized individuals through self-reporting, students might have engaged in a form of self-deceptive process which has been correlated with internal control beliefs and mental health conditions (e.g., Caputo, 2019a). Moreover, the current model mainly focused on “unconscious” factors as defense mechanism and alexithymia, it has been suggested that overwhelming affective stimulation may also lead to a disorganization of thought process. As such, cognitive schemas should be included in the relationship between PTSD from past trauma and distress for a more comprehensive model (Fang and Chung, 2018, 2019). Despite these limitations, the original contribution of this study is the empirical examination of the psychodynamic mechanisms (i.e., defensive organization and alexithymia) underpinning the relationship between PTSD and distress outcomes among university students. Those with PTSD who have alexithymic symptoms and use immature defenses may develop anxiety and thereby affect perceived quality of life (e.g., Martino et al., 2019b).

The present findings have some important clinical implications. First, screening for PTSD symptoms, alexithymia, and defense mechanisms followed by appropriate counseling is paramount for university students. Second, based on our findings, further studies need to be designed to investigate the effectiveness of psychodynamic treatments for PTSD and trauma among university students. These interventions should aim to empower students know more about their defense styles, explore their inner feelings, and get in touch with their internal self (Briere, 1996). The results of these studies will add to existing empirical and clinical evidence suggesting that psychodynamic approaches may increase the usage of mature defenses with concomitant decreased reliance on immature defenses in various trauma victims (e.g., Schottenbauer et al., 2008). Psychodynamic treatments could also be compared with non-invasive brain stimulation techniques for reducing trauma related anxiety. Such comparison might be of interest for scholars from the neuroscience community (Vicario et al., 2019).

## Conclusion

To conclude, following exposure to trauma, victims can develop PTSD affecting mental health. The severity of psychological distress was influenced by the degree of immature defenses used and their ability to identify, describe, and process distressing emotions.

## Data Availability Statement

The datasets generated for this study are available on request to the corresponding author.

## Ethics Statement

The studies involving human participants were reviewed and approved by the Survey and Behavioral Committee of the Chinese University of Hong Kong. The patients/participants provided their written informed consent to participate in this study. The animal study was reviewed and approved by Survey and Behavioral Research Ethics Committee of the Chinese University of Hong Kong.

## Author Contributions

The authors made equally important contributions to the manuscript.

## Conflict of Interest

The authors declare that the research was conducted in the absence of any commercial or financial relationships that could be construed as a potential conflict of interest.
